# The abrupt onset of the modern South Asian Monsoon winds

**DOI:** 10.1038/srep29838

**Published:** 2016-07-20

**Authors:** Christian Betzler, Gregor P. Eberli, Dick Kroon, James D. Wright, Peter K. Swart, Bejugam Nagender Nath, Carlos A. Alvarez-Zarikian, Montserrat Alonso-García, Or M. Bialik, Clara L. Blättler, Junhua Adam Guo, Sébastien Haffen, Senay Horozal, Mayuri Inoue, Luigi Jovane, Luca Lanci, Juan Carlos Laya, Anna Ling Hui Mee, Thomas Lüdmann, Masatoshi Nakakuni, Kaoru Niino, Loren M. Petruny, Santi D. Pratiwi, John J. G. Reijmer, Jesús Reolid, Angela L. Slagle, Craig R. Sloss, Xiang Su, Zhengquan Yao, Jeremy R. Young

**Affiliations:** 1 Institute of Geology, CEN, University of Hamburg, Bundesstrasse 55, Hamburg 20146, Germany; 2 Department of Marine Geosciences, Rosenstiel School of Marine & Atmospheric Science, University of Miami, Miami FL 33149, USA; 3 Department of Geology and Geophysics, University of Edinburgh, Grant Institute, The King’s Buildings, West Mains Road, Edinburgh EH9 3JW, United Kingdom; 4 Department of Geological Sciences, Rutgers, The State University of New Jersey, 610 Taylor Road, Piscataway NJ 08854-8066, USA; 5 Geological Oceanography Division, CSIR-National Institute of Oceanography, Dona Paula Goa 403004, India; 6 International Ocean Discovery Program, Texas A&M University, Discovery Drive, College Station TX 77845, USA; 7 Instituto Portugues do Mar e da Atmosfera (IPMA), Divisão de Geologia e Georecursos Marinhos, Avenida de Brasilia 6, 1449-006 Lisboa, Portugal; 8 Centro de Ciencias do Mar (CCMAR), Universidade do Algarve, Faro, Portugal; 9 Dr. Moses Strauss Department of Marine Geosciences, The Leon H. Charney School of Marine Sciences, University of Haifa, Carmel 31905, Israel; 10 Department of Geosciences, Princeton University, Guyot Hall, Princeton NJ 08544, USA; 11 Department of Geological Sciences, California State University Bakersfield, 9001 Stockdale Highway, Bakersfield, CA 93311, USA; 12 Physical Properties Specialist, Ecole Nationale Superieure de Geologie, Universite de Lorraine, 2 rue du Doyen Marcel Roubault, Vandoeuvre-les-Nancy 54501, France; 13 Petroleum and Marine Research Division, Korea Institute of Geoscience & Mineral Resources (KIGAM), Gwahang-no 124, Yuseong-gu, Daejeon 305-350, Korea; 14 Graduate School of Natural Science and Technology, Okayama University, 3-1-1 Tsushima-naka 700-8530, Japan; 15 Instituto Oceanográfico da Universidade de São Paulo, Praça do Oceanográfico, 191, São Paulo, SP 05508-120, Brazil; 16 Istituto di Scienze della Terra, Università di Urbino, Via S. Chiara 27, Urbino 61029, Italy; 17 Department of Geology and Geophysics, Texas A&M University, Mail Stop 3115, College Station TX 77843-3115, USA; 18 Department of Environmental Engineering for Symbiosis, Soka University, 1-236 Tangi-cyo, Hachioji-shi Tokyo 192-0003, Japan; 19 Graduate School of Science and Engineering, Yamagata University, 1-4-12 Kojirakawa-machi, Yamagata City 990-8560, Japan; 20 Environmental Science and Policy Department, David King Hall Rm 3005, MSN 5F2, George Mason University, University Drive, Fairfax, VA 22030-4444, USA; 21 Department of Geosciences, Geotechnology and Materials Engineering for Resources, Akita University, 1-1 Teagata-Gakuencho, Akita 010-8502 Japan; 22 Department of Earth and Life Sciences, Vrije Universiteit Amsterdam, De Boelelaan 1085, HV Amsterdam, The Netherlands; 23 Lamont-Doherty Earth Observatory, Columbia University, Borehole Bldg. 61 Route 9W, Palisades NY 10964, USA; 24 Earth and Environmental Sciences, University of Technology Queensland, R-Block 317, 2 George Street, Brisbane Queensland 4001, Australia; 25 Key Laboratory of Marginal Sea Geology, South China Sea Institute of Oceanology, Chinese Academy of Sciences, West Xingang Road, Guangzhou 510301, P.R. China; 26 Department of Marine Geology, First Institute of Oceanography (FIO) State Oceanic Administration (SOA), #6 Xian Xia Ling Road, Qingdao Shandong Province 266061, P.R. China; 27 Laboratory for Marine Geology, Qingdao National Laboratory for Marine Science and Technology, Qingdao, P.R. China; 28 Department of Earth Sciences, University College London, Gower Street, London WC1E 6BT, United Kingdom

## Abstract

The South Asian Monson (SAM) is one of the most intense climatic elements yet its initiation and variations are not well established. Dating the deposits of SAM wind-driven currents in IODP cores from the Maldives yields an age of 12. 9 Ma indicating an abrupt SAM onset, over a short period of 300 kyrs. This coincided with the Indian Ocean Oxygen Minimum Zone expansion as revealed by geochemical tracers and the onset of upwelling reflected by the sediment’s content of particulate organic matter. A weaker ‘proto-monsoon’ existed between 12.9 and 25 Ma, as mirrored by the sedimentary signature of dust influx. Abrupt SAM initiation favors a strong influence of climate in addition to the tectonic control, and we propose that the post Miocene Climate Optimum cooling, together with increased continentalization and establishment of the bipolar ocean circulation, i.e. the beginning of the modern world, shifted the monsoon over a threshold towards the modern system.

The South Asian Monson (SAM) is a seasonal reversal of winds accompanied by changes in precipitation with heavy rain during the summer monsoon. It is one of the most intense annually recurring climatic elements and of immense importance in supplying moisture to the Indian subcontinent thus affecting human population and vegetation, as well as marine biota in the surrounding seas. The seasonal precipitation change is one of the SAM elements most noticed on land, whereas the reversal of the wind regime is the dominating driver of circulation in the central and northern Indian Ocean realm. In the Indian Ocean, this monsoonal circulation and its attendant oxygen minimum were emplaced during the Neogene^1^ (Fig. 1A). There is, however, an ongoing discussion about several critical aspects. These include (1) the timing of the monsoon intensification, which has been placed at ~7 to 8 Ma^1^ or at ~28.7–22 Ma^2^,^3^,^4^,^5^, and therefore its relation to the high topographic relief linked to the uplift of the Tibetian-Himalayan orogeny^2^,^6^; (2) the linkage between the monsoon and the evolution of global cooling^7^; and (3) the interpretation of the establishment and fluctuations of the oxygen minimum zone (OMZ), as triggered by atmospheric circulation linked to the summer monsoon winds^1^.

New data acquired during IODP Expedition 359 from the Inner Sea of the Maldives provide a previously unread archive that reveals an abrupt onset of the SAM-linked circulation pattern and its relationship to the long term Neogene climate cooling. In particular it registers ocean current fluctuations and changes of intermediate water mass properties for the last 25 myrs that are directly related to the monsoon. Data also reveal how the Maldives carbonate platform responded to the Early and early Middle Miocene sea level fluctuations through changes of the platform growth pattern and growth interruptions.

## Results and Interpretations

Data collected during IODP Expediton 359 pertinent to the discussion of the SAM include stratigraphical, geophysical, and geochemical data that are displayed in Figs 2, 3, 4 and Tables 1 and 2. These data are described in detail below, followed by an interpretation.

**Figure 1:**
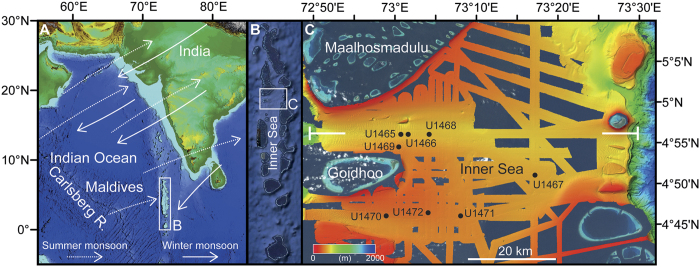
(**A**) Location of the Maldives with main wind directions. (**B**) Inner Sea with two rows of atolls. (**C**) Satellite and multibeam imagery with position of IODP Expedition 359 sites. The seaway connecting the Inner Sea with the Indian Ocean formed through the demise of two N-S oriented continuous carbonate banks. Demise occurred in steps, as shown by the occurrence of drowned relict banks. The tear-drop shapes of the drowned atolls indicate the presence of a west-directed current flowing through the newly formed marine passage^8^. White lines: position of seismic line shown in Figs 2 and S1. Maps were produced using the program Esri ArcMap 10.1 (www.esri.com). Bathymetric data in A and B were exported as Geotiffs from the application GeoMapApp 3.6.0 (www.geomapapp.org). In C, Worldwind satellite images (http://worldwind.arc.nasa.gov/java) were merged with multibeam data acquired during the cruises M74/4 and SO236.

The Maldives archipelago is a carbonate edifice that is elevated between 2000 and 4000 m above the surrounding seafloor, and therefore shielded from riverine terrestrial input (Fig. 1B,C). It contains a north-south–oriented double row of reef-rimmed atolls that enclose the up to 500 m deep Inner Sea, which is a natural sediment trap with continuous carbonate deposition. Together with the surrounding banks the Inner Sea provides a tropical record of Neogene sea-level changes and changes of the ocean current regime.

**Figure 2:**
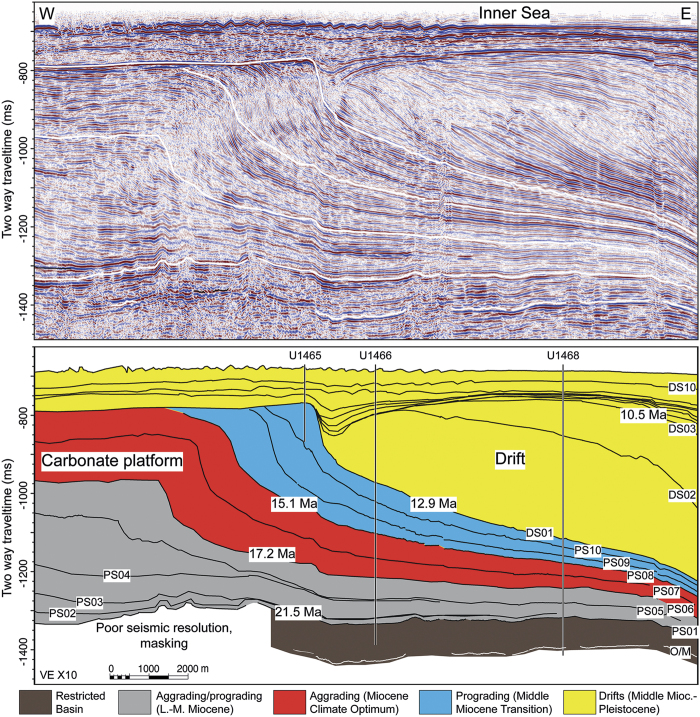
Seismic line and sequence stratigraphic interpretation of the Inner Sea illustrates the abrupt onset of current-controlled drifts (yellow) overlying a package of sea-level controlled carbonate platform strata. PS and DS denote the sequence boundaries defined in the platform and in the drift succession respectively. Three carbonate platform growth packages are differentiated: a lower to middle Miocene aggrading to prograding package, a dominantly aggrading middle Miocene package followed by a Middle Miocene prograding package. This seismic line provides a detail of a line cross-cutting the entire Inner Sea of the Maldives (Figure S1).

**Figure 3:**
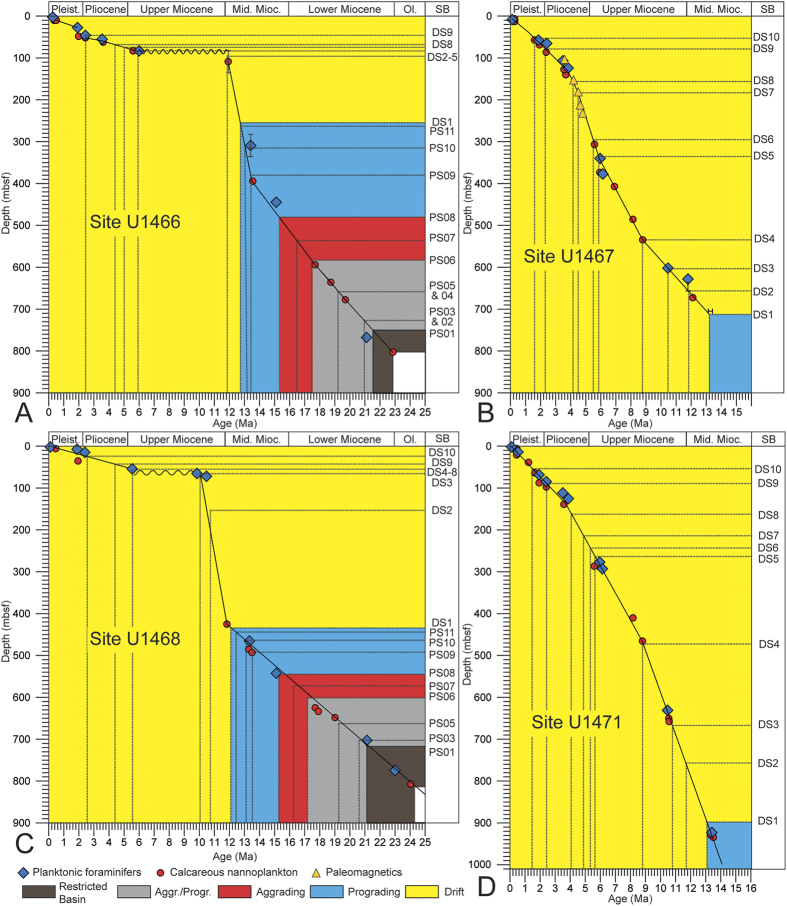
(**A**–**D**) Age-depths plots for the sites used in this study. The age of the switch from sea-level controlled platform growth (blue) to the current-dominated sedimentation (yellow) varies in a narrow range of 13.1 to 12.8 Ma with an outlier of 12.2. Ma at Site U1468 Ma. 12.9 Ma is used herein as a median age value. The beginning and the end of platform growth during the Miocene Climate Optimum is dated at ~17.2 Ma and ~15.1 Ma, respectively. Planktonic foraminifer and calcareous nannoplankton datums are listed in Tables S1 and S2 (Methods, Supplementary Materials). Colors refer to the sediment packages differentiated in Figs 2 and S1.

The modern currents in the Maldives are directly linked to the seasonal SAM-driven reversing winds (Fig. 1A), which are directed westward in the winter and eastward in the summer thus depositing major sediment drift bodies^8^,^9^,^10^. Seismically, the drift deposits are identified as convex outward prograding clinoforms that onlap and bury a succession of platform carbonates (Figs 2 and S1). The base of these current deposits are herein used to date the onset of the regional wind system which is nowadays associated with the SAM. The underlying carbonate platforms display prograding pulses that are related to sea-level fluctuations^9^ and the global climate evolution during the past 25 myrs (Figs 2 and S1). Changes in the interplay of these two processes produced breaks in the sedimentary pattern like interruptions of platform growth during sea-level lowerings. Surfaces that delimit packages with distinct growth characteristics are sequence boundaries, and their ages, thus, mark the turning points in the sedimentary evolution. Biostratigraphic data obtained during IODP Expedition 359 provide precise ages of these sequence boundaries (Fig. 3, Tables 1 and 2).

**Figure 4:**
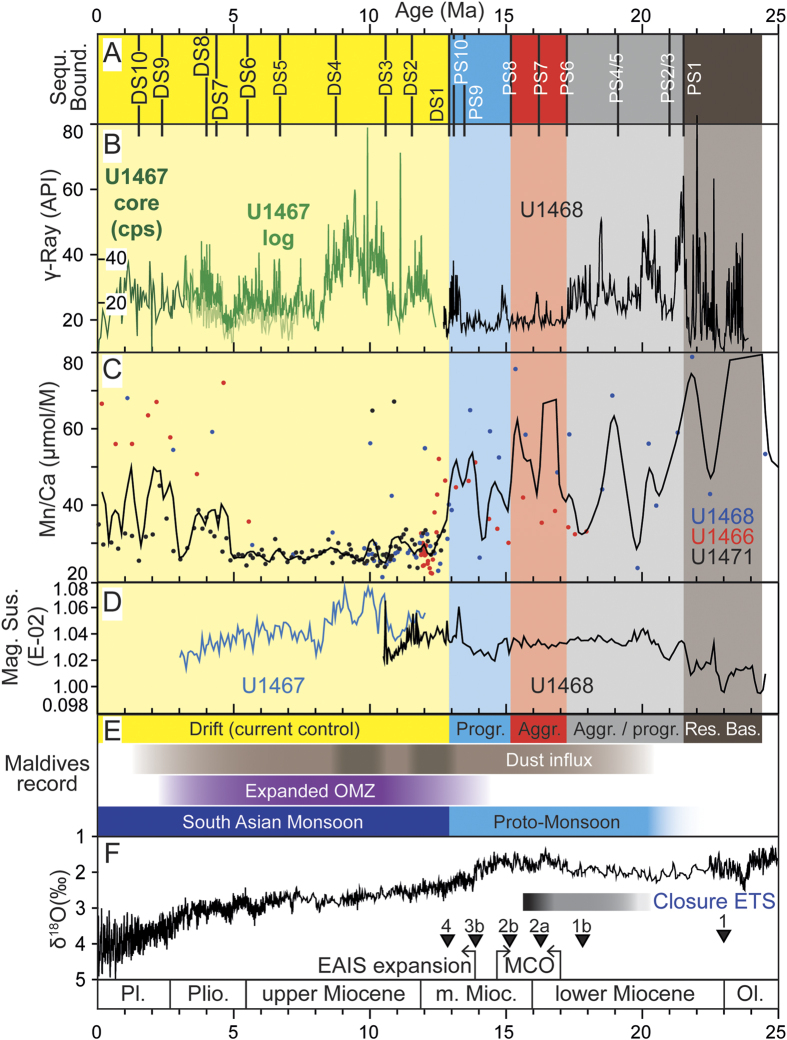
(**A**) Age of sequence boundaries; (**B**) total gamma ray in downhole logs for Sites U1467 and U1468. For Site U1467, core measurements were used for the upper interval of the succession (no downhole logs available). (**C**) Mn/Ca ratios at Sites U1466, U1468, and U1471. Black line shows a 3 point running average. (**D**) Magnetic susceptibility from downhole logs at Sites U1467 and U1468. (**E**) Stratigraphic breaks and changes, variations of the dust influx and of the OMZ and timing of SAM and Proto-Monsoon as proposed in this study. (**F**) Deep sea oxygen isotope record^9^, timing of the eastern Antarctic ice sheet (EAIS) expansion^30^, timing of the middle Miocene climate optimum (MCO)^31^, Mi-events 1–4^12^, and restriction^32^ as well as final closure of the eastern Tethys seaway^33^.

Examining the Maldives sedimentary system prior to the onset of the drift deposits indicates that sea-level fluctuations were the dominant agents in the sediment distribution and that monsoon related features were subordinate. The Maldives architecture with two N–S running carbonate banks was established during the Oligocene^11^. Shrinking of the banks to a width of around 5 km ~ 24 Ma ago preceded an apparent restriction of the Inner Sea area in which sapropel-like organic rich layers, with total organic contents (TOC) of up to ~7% alternating with chalks are deposited between ~24 and ~21.5 Ma. High gamma ray values reflect TOC enriched intervals (Fig. 4B). Sapropel formation started at the Mi-1 event (ca. 23 Ma), an episode interpreted as substantial glaciation^12^. From ~21.5 to ~17.2 Ma, the western carbonate banks increased in width through bank aggradation and eastward progradation of the bank edge into the Inner Sea (Figs 2 and S1). The eastern banks during this time did not widen^11^, but were segmented between ~19 and ~17.2 Ma through demise of banks thereby opening the Inner Sea to the Indian Ocean in the east and reducing the restriction of the basin (Fig. 1B)^10^. This resulted in an overall reduction of organic matter deposition, although some intervals with higher organic contents remained (Fig. 4B). At ~17.2 Ma, i.e. at the sequence boundary (SB) of PS6, the aggrading/prograding platform growth changed to a predominantly aggradational mode in sequences PS6 and PS7 (~15.1 Ma) that is typical for the formation under a rising sea level. A turning point in platform reorganization occurred at the base of PS8 at ~15.1 Ma when the platform sequences PS8–PS10 stack in front of each other (Fig. 2). These prograding sequences reflect platform growth under an overall lowered sea level.

This platform evolution is clearly linked to global climate and sea level evolution^12^,^13^,^14^ (Fig. 4). The aggrading PS6 and PS7 package formed during the Miocene Climate Optimum (MCO) (Fig. 4F) with a high eustatic sea level, the start of the progradation falls within the time of the transition towards a cooler climate, with a global sea-level lowering and the expansion of the East Antarctic Ice Sheet which began at 13.9 Ma reflected by the Mi-3b event which in the Maldives coincides with the SB of PS9. This tracing of the global sea level evolution ended at ~12.9 Ma when ocean currents started to control the sedimentary system.

We deduce the wind pattern that likely is related to weak proto-monsoon system between 24 and 12.9 Ma from two lines of evidence. First, the eastward progradation of the platforms (Fig. 2) indicates westerly winds, i.e. summer monsoon winds, because carbonate platform progradation occurs preferentially towards the leeward side^15^. Second, the magnetic susceptibility record from the Inner Sea sediments following^16^ is proposed to reflect eolian dust input (Fig. 4D). This flux from India and Asia is generally low and linked to the winter monsoon winds^17^, which transport dust as far as 5°S^18^. The investigated area, now positioned between 4.5°–5°N, was at 5°S at around 31 Ma and crossed the equator around 20 Ma^19^. Magnetic susceptibility rises stepwise in deposits formed between ~24 and ~20 Ma with peaks of high values at ~22.6 and ~21.5 Ma, followed by a stabilization from ~20.5 Ma until 13.5 Ma (Fig. 4D).

The major and abrupt turning in the sedimentary and geochemical record occurs at ~12.9 Ma (Fig. 4). The sea-level controlled platform growth was greatly reduced leading to the demise of some Maldives platforms^8^,^9^,^10^. This partial drowning went hand in hand with the onset of current-dominated deposition of drift sequence DS 1 (Fig. 2). The depositional turnover is along the same seismic reflection throughout the entire Inner Sea, documenting widespread and coeval occurrence of drift deposits^8^,^9^,^10^ (Figure S1). This profound change in the depositional dynamics is the direct physical evidence of the onset of vigorous wind-driven currents with the dominance of westerlies, as is also the case for the modern summer monsoon winds in the Maldives^9^,^10^,^11^.

Platform drowning seems to be the combined product of change in sea level and ocean circulation. As sea level started to fall from the height of the MCO, platforms were repeatedly exposed. During the subsequent sea-level rises strong currents swept the platforms^8^, thus preventing the re-establishment of shallow-water ecosystems and causing the demise of several platform segments. A concomitant higher particulate organic matter (POM) accumulation (Fig. 4B) shows initiation of increased upwelling. Today the Maldives act as a flow barrier to the reversing wind-driven currents, triggering upwelling cells downstream of the atolls with high primary productivity^20^ causing higher chlorophyll a concentrations during the winter monsoon flow^21^. Thus, the variations in the organic contents in the Inner Sea sediments of DS1 likely record the onset and intensity fluctuations of this upwelling.

The increase in POM content occurs in concert with an expansion of the Indian OMZ. Wind-driven upwelling during the summer monsoon generates the OMZ in the Arabian Sea, and the extent of this zone over geological time reflects variations in atmospheric and oceanic circulation^1^. The investigated part of the Inner Sea lies at 300 and 500 m water depth and is within the OMZ. We use fluctuations in the Mn concentration to trace variations of the OMZ through time, since the flux of Mn into sediment forming within the OMZ is reduced, because of elevated solubility of Mn in O_2_-deficient waters^22^. Between 25 and ~12.5 Ma the Mn/Ca ratio shows a trend to decreasing values with some outliers (Fig. 4C). At ~12.9 Ma the Mn/Ca ratio shows a general drop, punctuated by short excursions towards higher values up to 10 Ma. The expanded OMZ appeared fairly stable between 10 and ~5 Ma. In younger deposits, Mn/Ca ratio fluctuations record variations of the OMZ extension and finally a retraction beyond the Maldives after ~0.8 Ma. Whereas OMZ expansion and POM content increase seem to be coupled, POM content decreases at ~8 Ma, a time with a stable and extended OMZ. The independent physical stratigraphic record and geochemical lines of evidence from cores of the Inner Sea thus unequivocally place the onset of the SAM circulation at ~12.9 Ma. This age was previously proposed for the SAM wind system onset^23^, based on the stable isotope analysis of planktonic foraminifera in the Arabian Sea.

Magnetic susceptibility increases in Maldives deposits formed between ~13.5 to ~11 Ma, and reach maximum values in ~11 to ~8.1 Ma old sediments. Deposits younger than ~8.1 Ma display lower magnetic susceptibility values and start to decrease at ~6 Ma. This decrease of the magnetic susceptibility values parallels a change in drift architecture beginning in DS 5 at~ 6.7 Ma (Fig. 2). In older sequences, small west-directed drifts are observed at the eastern side of the Inner Sea but these became inactive at ~6.7 Ma and moats along the atoll edges developed reflecting the onset of a N to NE directed bottom current (Figure S1).

## Discussion

Together, the direct physical record in the drift deposits and the proxies retrieved in the cores provide a robust chronology of the evolution of the current pattern on the Maldives which nowadays is controlled by the winds of the SAM. Beginning at 24 Ma the evolution and the controlling factors of the Neogene SAM can be drawn as follows. The stepwise increase of dust deposition to the Maldives between 24 and 20 Ma encompasses the time window for the Neogene Asian desertification from loess deposits in China at 22 Ma^3^, with first sediments indicative of widespread arid conditions at 25 Ma^4^ and ~28.7–22.6 Ma respectively^5^. Modeling indicate that this desertification after the retreat of the Paratethys further facilitated the shift from a temperate to a continental central Asian climate^24^,^25^ therefore providing a source of eolian dust. A stable and continuous dust input is achieved at 20 Ma (Fig. 4D,E). During this stage, winds in the Maldives caused the eastward progradation of the western atolls but were not strong enough to generate drift deposits. This changed with the sudden strengthening of the wind and resulting currents at 12.9 Ma that resemble the modern strength of the monsoon system. Therefore, the circulation between ~20 and 12.9 Ma may be addressed as a proto-monsoon to differentiate it from the vigorous wind system which abruptly established at ~12.9 Ma, as imaged by the onset of drift deposition in the Inner Sea. Magnetic susceptibility values also point towards a weaker dust export from the Indian and Asian mainland during this time interval.

The abrupt onset of the strong modern monsoon system at ~12.9 Ma is pervasive in our data set. The rates of changes in the SAM dynamics over a few 100 kyrs at best as revealed by our data clearly indicate that the onset of the modern monsoon winds occurred much too rapid to be solely explained by tectonic processes such as the uplift of the Tibetian-Himalayan Orogen^2^. A series of factors, including global paleoclimate, the distribution of the continents, closure of seaways, and the concomitant changes in the global circulation pattern are responsible for the observed pattern of weak proto-monsoon and the abrupt SAM strengthening. This intensification is also reconstructed from upwelling indicators in the Arabian Sea^23^.

This onset by ~900 kyrs postdates the end of the Middle Miocene climate transition^26^, i.e. the time of growth of the East Antarctic Ice Sheet (Fig. 4F), as well as of global and of deep water cooling (Fig. 4F). The deep-water circulation system at this time also developed a bi-polar aspect, when the North Atlantic end member became an important component, complementing the persistent southern source of deep water circulation^27^,^28^. The climate and circulation changes also coincide with the increased continentalization of climate and seasonality as well as the progressive closure of the Tethys seaway that resulted in increased atmospheric gradients and seasonal wind speeds^12^.

Therefore, we see the abrupt intensification of the summer monsoon related to stepping over a threshold that was created by the closure of the Tethys and the uplift of the Tibetian-Himalayan Orogen that created a strong seasonal atmospheric pattern and was compounded by global cooling and the onset of the oceanic and circulation during the late middle Miocene. The respective influence of each factor will have to be assessed by modeling that now is possible as the exact timing of the strengthening of the SAM is determined by this data set.

## Methods

### Seismics and hydroacoustics

Seismic signals were generated by two clustered GI-guns, each with a volume of 45 in^3^ for a 105-in^3^ generated injector volume. A digital 144-channel streamer array with an active length of 600 m and an asymmetric group interval was used during the cruises M74/4 with r/v METEOR (2007) and SO236 with r/v SONNE (2014). The data was digitized with seven SeaMUX 24 channel 24 bit digitizing modules, configured in six multiple arrays totaling 144 channels. The shot point distance during the entire cruise was 12.5 m. The dominant frequencies center around 100–120 Hz. Processing of reflection seismic data was done using the software package ProMAX 2D (Halliburton-Landmark). The data is processed to zero phase, filtered in time and f-k domain, and corrected for dip moveout. In basinal areas a suppression of multiple reflections was achieved by predictive deconvolution of pre-stacked data. Amplitude losses were compensated by a power function. Interpretation and visualization was done using the software package Petrel (Schlumberger). Depending on depth, the vertical resolution of the newly acquired data is approximately 4–6 m. Seismic interpretation was performed on time–migrated data in time domain. As the continuity of the reflections in part is weak, the instantaneous phase was also used for tracing. Seismic data were linked to core data using check shot surveys and downhole logging data.

Multibeam imaging was performed with a hull-mounted EM120 multibeam echosounder (Kongsberg Maritime) during the cruises M74/4 with r/v METEOR (2007) and SO236 with r/v SONNE (2014). The EM120 is a high-resolution sea-floor-mapping system with 256 simultaneous beams operating in the 12 kHz range, and covering a swath width of up to 5.5 times the water depth. The beams are stabilized for roll, pitch, and yaw. Data obtained was post-processed using the software package Neptune (Kongsberg Maritime). Visualization including gridding and refining of surfaces was done using the software packages Caris and Fledermaus (IVS 3D).

### Biostratigraphy

Calcareous nannofossils and foraminifera were studied at all sites in core catcher samples. Samples from core sections were also examined when a more refined age determination was necessary and when time permitted. Biostratigraphic events, mainly the first occurrence (FO; or base) and last occurrence (LO; or top) of the well-established diagnostic species, are tied to the geomagnetic polarity timescale of ref. 29. The depth of the events was calculated as the midpoint between the two samples that enclose each event. Applying the results of the check shot surveys, a direct correlation of the seismic stratigraphy and the biostratigraphy was achieved. Dating of the drift onset was a high-priority we cored the key interval at four sites. Key data is provided by the last occurrence of *Sphenolithus heteromorphus*, the last common occurrence of *Cyclicargolithus floridanus* and the first occurrence of *Fohsella fohsi*, each of which occur are ell-established as occurring at about 13.5 Ma. At all four sites these three events occur significantly below the base of the drift so this is a robust maximum age for the event. The minimum age cannot be tied down with similar certainty but our age models based on multiple events (Fig. 3) suggested a range of ages from 13.1 to 12.8 Ma, with one outlier age of 12.2 Ma. The outlier was site U1468, which was near the basin centre where the onset of drift deposition may have been delayed and at which sandwaves may have affected the base of the drift. So we have adopted a median age of 12.9 Ma, with an uncertainty of ca. +/−0.4 Ma.

### Downhole Logging

The Hostile Environment Natural Gamma Ray Sonde (HNGS) which was deployed during downhole wireline logging uses two bismuth germanate scintillation detectors and five-window spectroscopy to determine concentrations of potassium (in weight percent), thorium (in parts per million), and uranium (in parts per million) from the characteristic gamma ray energies of isotopes in the 40 K, 232Th, and 238U radioactive decay series, which dominate the natural radiation spectrum. At Expedition 359 Sites U1467 and U1468, spectral gamma ray data show that uranium is the primary contributor to variability in total gamma ray. Variations in the total gamma ray record shown in Fig. 4B can therefore be interpreted as changes in uranium and therefore only the total gamma ray intensity is shown in Fig. 2A.

The Magnetic Susceptibility Sonde (MSS) is a nonstandard wireline tool designed by Lamont-Doherty Earth Observatory (LDEO). It measures the ease with which formations are magnetized when subjected to a magnetic field. The ease of magnetization, or susceptibility, is ultimately related to the concentration and composition (size, shape, and mineralogy) of magnetic minerals in the formation. The MSS dual-coil sensor provides ~40 cm resolution measurements, with ~20 cm depth of horizontal investigation. Although relative changes in susceptibility are likely accurate, the absolute values measured by the magnetic susceptibility tool are still in the process of being calibrated and should be treated with caution. The MSS was run as the lowermost tool in the triple combo tool string, using a specially developed data translation cartridge to enable the MSS to be run in combination with the Schlumberger tools. Magnetic susceptibility data from the MSS are plotted as uncalibrated units multiplied by a factor of 102 (Fig. 4D). Although relative changes in susceptibility are likely accurate, the absolute values measured by the magnetic susceptibility tool should be treated with care.

### Physical properties

Whole-round cores were measured with the Natural Gamma Radiation Logger using a system designed and built at the Integrated Ocean Drilling Program-US Implementing Organization (USIO) (Texas A&M University, USA). A measurement run consisted of two sample positions, 10 cm apart, for a total of 16 measurements per 150 cm section. Counting time was chosen as 5 min per position, or ~10 min per core, yielding statistically significant energy spectra. Natural gamma ray data measured on whole round cores are reported in counts per second (cps).

### Geochemistry

The samples used for the Mn/Ca analyses were obtained from the interstitial water squeezed cakes and in instance where no material was available, from the head space samples. The samples were freeze dried and 10 mg of ground sample dissolved in equal amounts (5 cm3) of 2% acetic acid and 2% nitric acid. After digestion the solution was centrifuged and the supernatant removed for analysis using a Leeman ICP-AES. Standardization was achieved using three solutions to which varying amounts of stock Mn solution had been added and which contained equivalent concentrations of calcium and magnesium to the samples.

## Additional Information

**How to cite this article**: Betzler, C. *et al*. The abrupt onset of the modern South Asian Monsoon winds. *Sci. Rep.* **6**, 29838; doi: 10.1038/srep29838 (2016).

## Supplementary Material

Supplementary Information

## Figures and Tables

**Table 1:** Calcareous nannofossil events considered for Expedition 359.

**Event**	**Age (Ma)**
FO *Emiliania huxleyi*	0.29
LO *Pseudoemiliania lacunosa*	0,44
LO *G. lumina*/ small *Gephyrocapsa* interval	1,24
LO *Calcidiscus macintyrei*	1,6
LO *Discoaster brouweri*	1,93
LO *Discoaster pentaradiatus*	2,39
LO *Sphenolithus abies*	3,54
LO *Reticulofenestra pseudoumbilicus*	3,7
LO *Discoaster quinqueramus*	5,59
FO *Nicklithus amplificus*	6.91
FO *Discoaster berggrenii/quinqueramus*	8,29
FO base small *Reticulofenester* interval	8,79
LO *Discoaster hamatus*	9,53
FO *Discoaster hamatus*	10,55
LO *Coronocycus nitescens*	11.9
LCO *Cyclicargolithus floridanus*	13.28
LO *Sphenolithus heteromorphus*	13,53
FO *Sphenolithus heteromorphus*	17,71
LO *Sphenolithus belemnos*	17,95
FO *Sphenolithus pseudoheteromorphus*	18.75
FO *Sphenolithus belemnos*	19,03
LO *Sphenolithus pseudoheteromorphus*	19.72
FO *Sphenolithus delphix*	22.82
LO *Sphenolithus delphix*	23,11
LO *Sphenolithus ciperoensis*	24,04

Ages are based on ref. 29. FO: first occurrence; LO: last occurrence.

**Table 2:** Planktonic foraminifer datum events considered to reconstruct the chronology of Expedition 359 sediment cores.

**Datum**		**Age (Ma)**	**Zone/Subzone**
LO	*Globigerinoides ruber* (pink)	0,12	PT1b
LO	*Globorotalia tosaensis*	0,61	PT1b/PT1a
LO	*Globigerinoides fistulosus*	1,88	PL6/PT1a
LO	*Globorotalia limbata*	2,39	PL5
FO	*Globigerinoides fistulosus*	3,33	PL5
LO	*Dentoglobigerina altispira*	3,47	PL4/PL5
LO	*Globorotalia margaritae*	3,85	PL2/PL3
FO	*Sphaeroidinella dehiscens sensu lato*	5,53	PL1
FO	*Globorotalia tumida*	5,57	M14/PL1
LO	*Globoquadrina dehiscens*	5,92	M14
LO	*Globorotalia lenguaensis*	6,14	M13b/M14
FO	*Neogloboquadrina acostaensis*	9,83	M12/M13a
LO	*Paragloborotalia mayeri*	10,46	M11/M12
LO	*Fohsella fohsi*	11,79	M9b/M10
FO	*Fohsella fohsi*	13,41	M9a/M8
FO	*Orbulina suturalis*	15,1	M5b/M6
LO	*Paragloborotalia kugleri*	21,12	M1b/M2
FO	*Paragloborotalia kugleri*	22,96	Oligocene/M1a

Ages are based on ref. 29. FO: first occurrence; LO: last occurrence.
