# Supplementary material for: The abrupt onset of the modern South Asian Monsoon winds
Source: Sci Rep. 2016 Jul 20;6:29838. doi: 10.1038/srep29838 (PMC4951686; doi:10.1038/srep29838)
Supplement: Supplementary Information [file srep29838-s1.pdf]

The abrupt onset of the modern South Asian Monsoon winds

Authors:

Christian Betzler[1], Gregor P. Eberli[2], Dick Kroon[3], James D. Wright[4], Peter K. Swart[2], Bejugam Nagender Nath[5], Carlos A. Alvarez-Zarikian[6], Montserrat Alonso-García[7], Or M. Bialik[8], Clara L. Blättler[9], Junhua Adam Guo[10], Sébastien Haffen[11], Senay Horozal[12], Mayuri Inoue[13], Luigi Jovane[14], Luca Lanci[15], Juan Carlos Laya[16], Anna Ling Hui Mee[2], Thomas Lüdmann[1], Masatoshi Nakakuni[17], Kaoru Niino[18], Loren M. Petruny[19], Santi D. Pratiwi[20], John J.G. Reijmer[21], Jesús Reolid[1], Angela L. Slagle[22], Craig R. Sloss[23], Xiang Su[24], Zhengquan Yao[25], Jeremy R. Young[26].

Supplementary Information:

Figure S1: Seismic line of the Inner Sea of the Maldives.

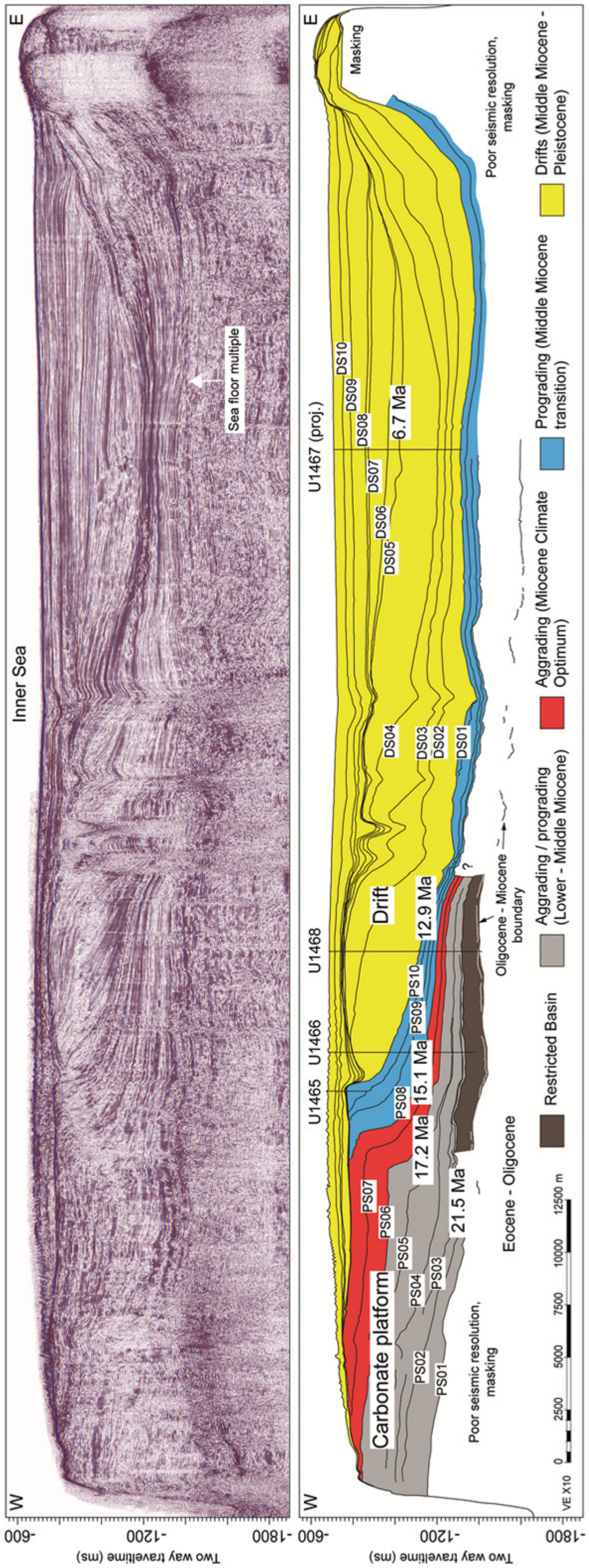

Supplementary Figure S1

Seismic lines (see Figure 2 for position) with location of IODP Expedition 359 drill sites and position of sequence boundaries.
